# Publisher Correction: A neural m^6^A/Ythdf pathway is required for learning and memory in *Drosophila*

**DOI:** 10.1038/s41467-021-22219-8

**Published:** 2021-03-15

**Authors:** Lijuan Kan, Stanislav Ott, Brian Joseph, Eun Sil Park, Wei Dai, Ralph E. Kleiner, Adam Claridge-Chang, Eric C. Lai

**Affiliations:** 1 Department of Developmental Biology, Sloan-Kettering Institute, New York, NY USA; 2 Program in Neuroscience and Behavioral Disorders, Duke-NUS Medical School, Singapore, Singapore; 3 Louis V. Gerstner, Jr. Graduate School of Biomedical Sciences, Memorial Sloan Kettering Cancer Center, New York, NY USA; 4 Department of Chemistry, Princeton University, Princeton, NJ USA; 5 Institute for Molecular and Cell Biology, A*STAR, Singapore, Singapore; 6 Department of Physiology, National University of Singapore, Singapore, Singapore

**Keywords:** RNA modification, Short-term memory

Correction to: *Nature Communications* 10.1038/s41467-021-21537-1, published online 05 March 2021.

The original version of the Supplementary Information associated with this Article contained an error in Supplementary Data 2 in which information that is not pertaining to the data reported in this paper was inadvertently included. The HTML has been updated to include a corrected version of Supplementary Data 2.

## Supplementary information

Supplementary Data 2

