# Supplementary material for: A neural m6A/Ythdf pathway is required for learning and memory in Drosophila
Source: Nat Commun. 2021 Mar 5;12:1458. doi: 10.1038/s41467-021-21537-1 (PMC7935873; doi:10.1038/s41467-021-21537-1)
Supplement: Supplementary file 1 — Supplementary Information [file 41467_2021_21537_MOESM1_ESM.pdf]

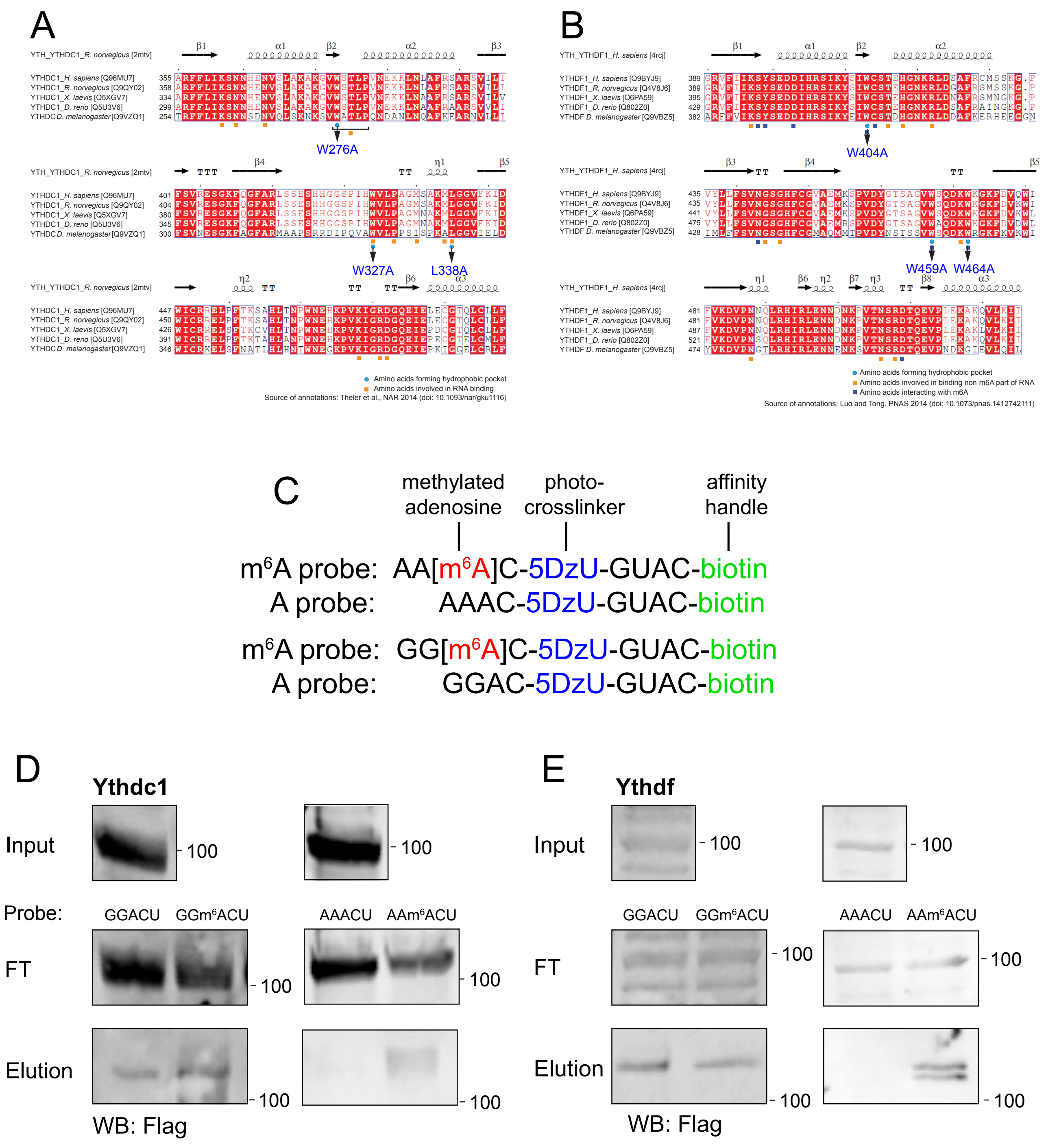

Supplementary Figure 1. m^{6}A binding properties of *Drosophila* YTH proteins.

(A-B) YTH domains from nuclear Ythdc1 (A) and cytoplasmic Ythdf (B) are homologous between *Drosophila* and vertebrates. The critical tryptophan/leucine residues in the Ythdc1/Ythdf m^{6}A binding pocket were mutated into alanines, as marked with arrows. (C) Sequences of unmodified and modified RNA probes used for photo-crosslinking assays with YTH proteins. (D) Ythdc1 showed modestly enhanced association to GGm^{6}ACU vs. GGACU probes, but showed clearly preferential crosslinking to AAm^{6}ACU compared to AAACU probes. (E) Ythdf did not exhibit preferential association to GGm^{6}ACU vs. GGACU probes, but was specifically crosslinked to AAm^{6}ACU relative to AAACU probes.

Supplementary Figure 1

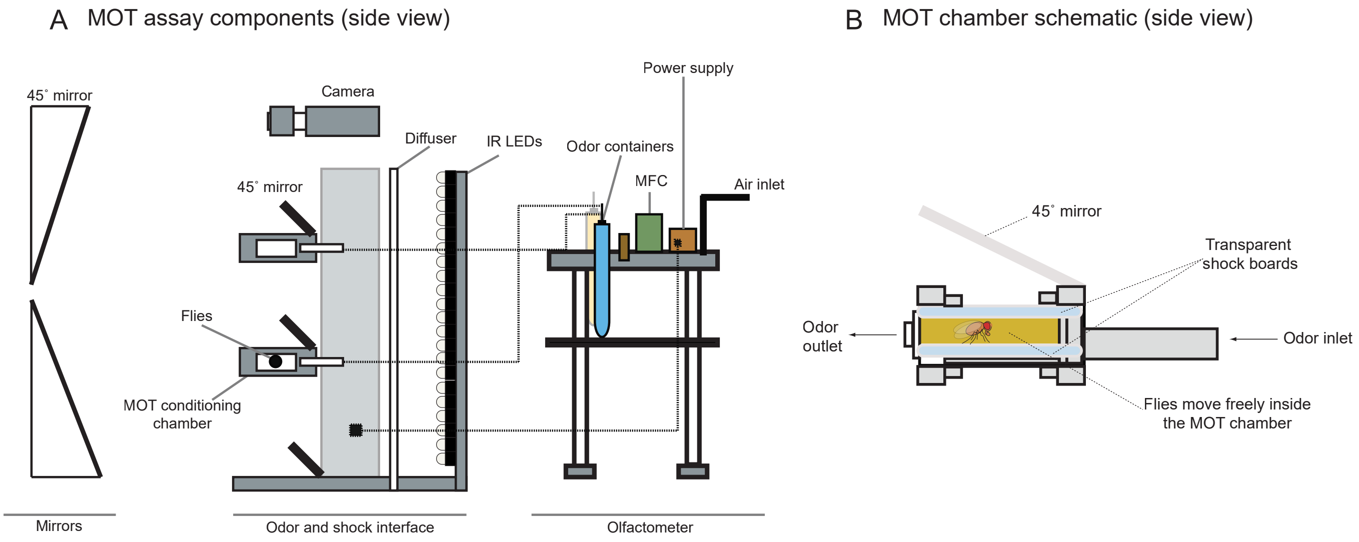

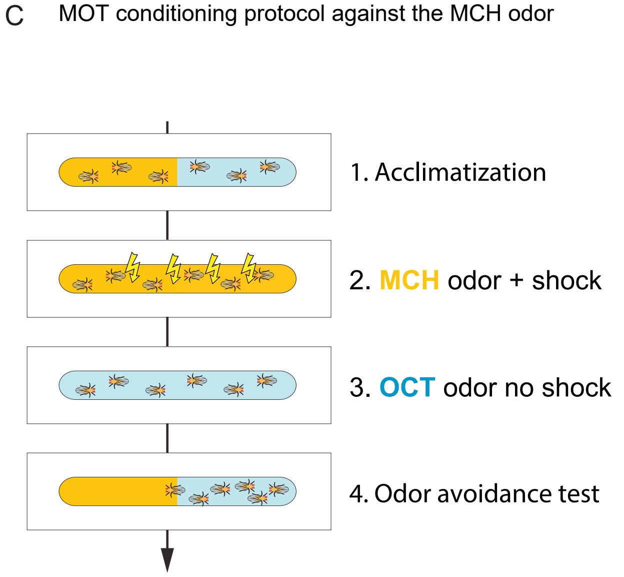

D Complete conditioning protocol

Conditioning sequence	Phase	Phase duration in min
Air	Acclimatization	1
Both odors	Bias test	2
Air	Acclimatization	1
Shock + odor A	Training	1
Air	Acclimatization	2
No shock + odor B	Taining	1
Air	Acclimatization	2
Both odors	Pre-agitation test	2
NA	Agitation	0.5
Both odors	Post-agitation test	2

Supplementary Figure 2. Structure and conditioning protocol of Multifly Olfactory Trainer (MOT) for short term memory evaluation.

(A) Overall schematic of MOT assay components. (B) Detailed schematic of the MOT chamber from the side view. (C) Sequence of a standard MOT conditioning protocol. After *Drosophila* were placed in the chambers and were given 60 s to acclimatize in pure air, one half of the chamber was exposed to MCH while the other half to OCT for during the bias test. After another 90 s of pure air, the whole arena was exposed for 60 s to the conditioned odor in the presence of a foot shock. This was followed by 90 s of air and the exposure of the whole arena to the second odor in the absence of shock. After 90 s of pure air, conditioning performance was tested for 120 s by exposing one side of the arena to the shock-associated odor and the other side to the neutral odor. (D) Complete conditioning protocol.

Supplementary Figure 2

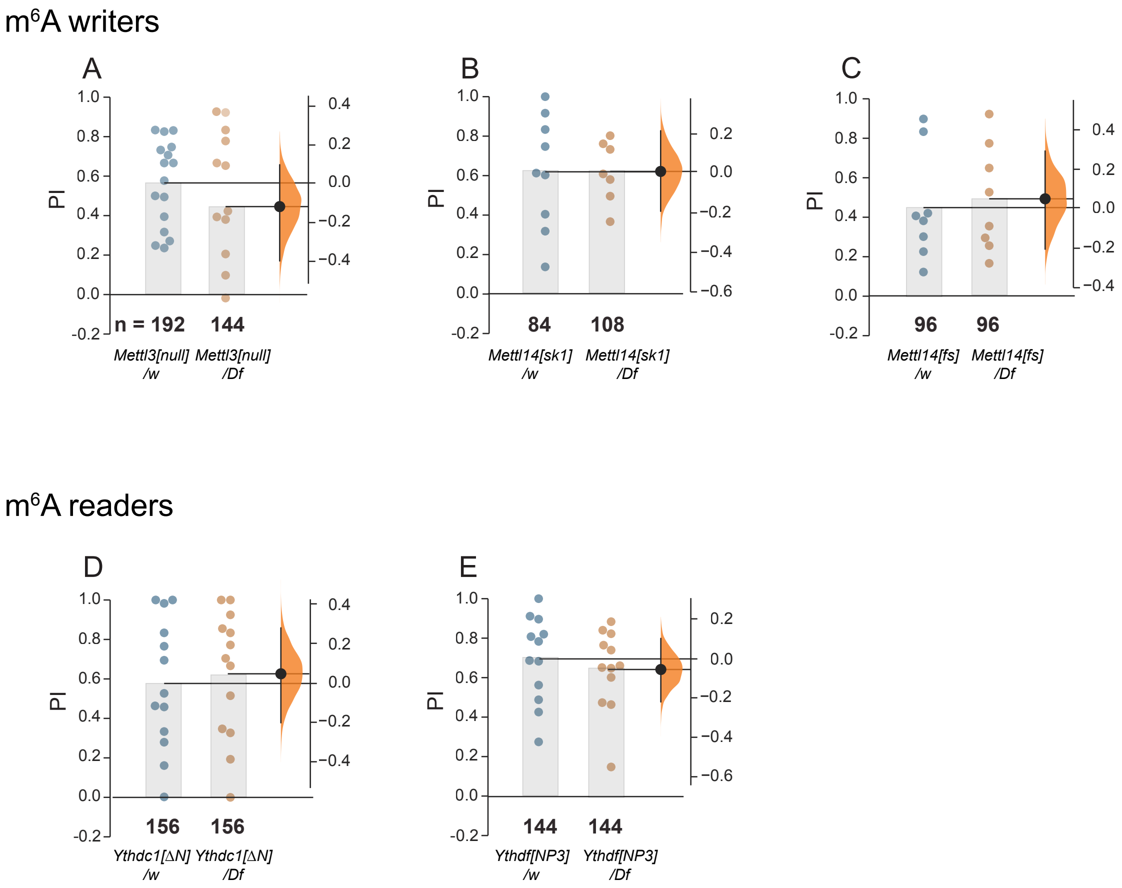

Supplementary Figure 3. Minimal impairment of STM in 10 day old m^{6}A pathway mutants.

(A) We observed a slight reduction in short term learning and memory (STM) in the odor avoidance paradigm in hemizygous *mettl3* mutants compared to heterozygous controls. *Mettl3[null]/Df* vs *Mettl3[null]/w* = -0.12[95CI -0.4, +0.1] p = 0.32. (B-E) No changes in STM performance were observed in other m6A pathway mutants. *Mettl14[sk1]/Df* vs *Mettl14[sk1]/w* = 0[95CI -0.2, +0.21] p = 0.9881. *Mettl14[fs]/Df* vs *Mettl14[fs]/w* = 0.04[95CI -0.21, +0.29] p = 0.7494. *Ythdc1[ΔN]/Df* vs *Ythdc1[ΔN]/w* = 0.05[95CI -0.2, +0.28], p = 0.706. *Ythdf[NP3]/Df* vs *Ythdf[NP3]/w* = -0.06[95CI -0.25, +0.18], p = 0.5961. The data were analyzed from the total number of fly shown in the bars and expressed as mean. All control–test differences are displayed as effect sizes with error curves and 95% confidence intervals, two-tailed Mann-Whitney P values are shown for legacy purposes only. Source data are provided as a Source Data file.

Supplementary Figure 3

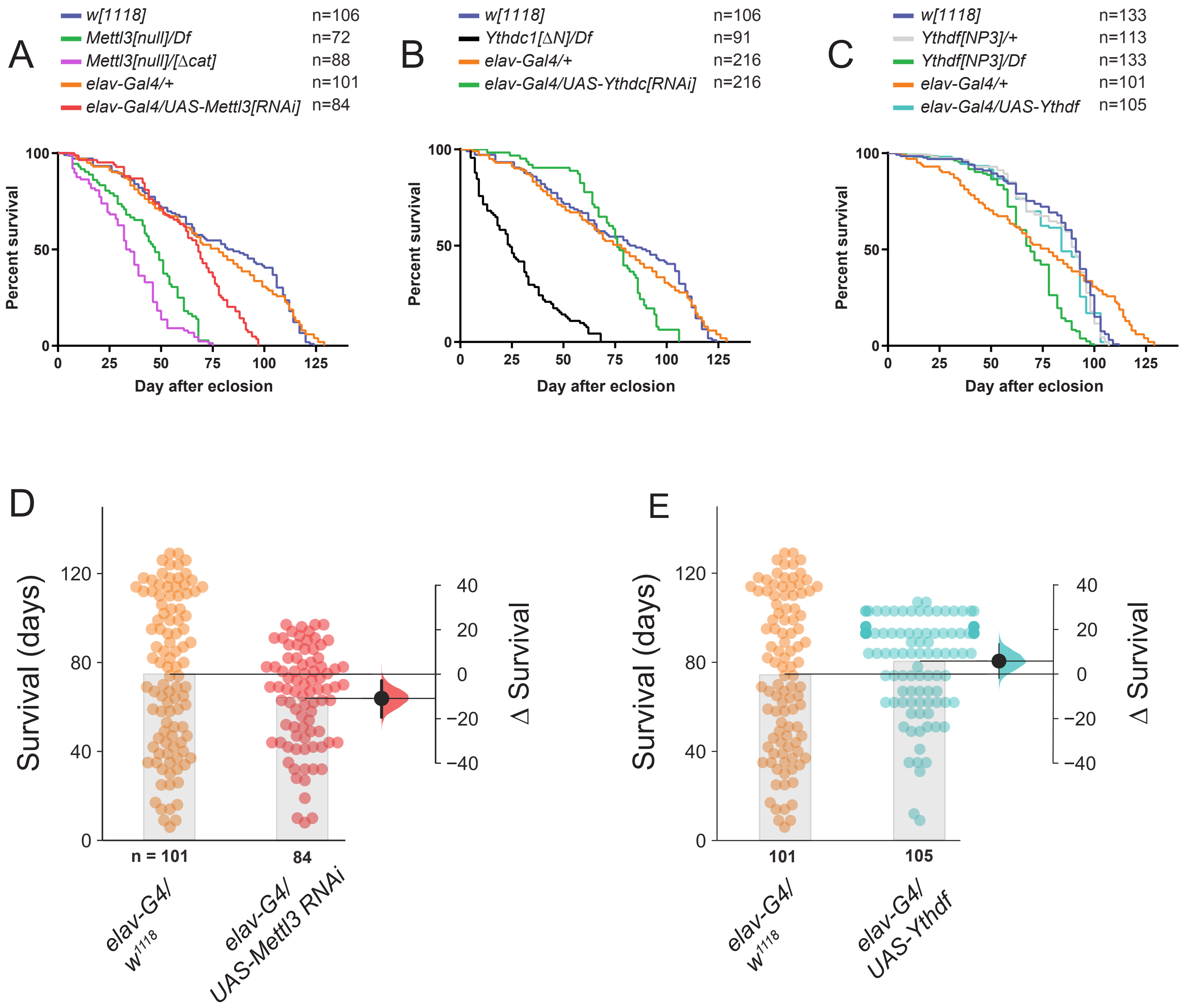

Supplementary Figure 4. Lifespan measurements of m6A pathway manipulations.

(A-C) Kaplan-Meier survival curves of adult *Drosophila* lifespan. These data correspond to scatter plots of adult survival shown in main Figure 3A-C. (A) Loss of *Mettl3* strongly impaired *Drosophila* survival as compared to heterozygous control or w1118 flies. Neuronal-specific depletion of Mettl3 led a mild reduction of lifespan. (B) Loss of *Ythdc1* strongly reduced survival. Neuronal-specific depletion of *Ythdc1* moderately reduced lifespan. (C) Loss of *Ythdf* moderately reduced survival in the hemizygous background as compared to heterozygotes and the *w[1118]* control. All survival experiments were performed at 23ºC and sample sizes for the respective genotypes are given in Figure 3. (D) Neuronal depletion of *Mettl3* using *elav-Gal4* and *UAS-Mettl3-RNAi* led to a mild reduction in lifespan compared to the *elav-Gal4* driver alone. *elav-G4/UAS-Mettl3-RNAi* vs *elav-G4/+* = -10.89[95CI -19.4, -2.9] p = 0.021. (E) Neuronal overexpression of Ythdf did not affect lifespan. *Elav-G4/UAS-Ythdf* vs *w[1118]* = 5.8[95CI -1,9, 13.7] p = 0.436. The data were analyzed from the total number of fly shown in the bars and expressed as mean. All control–test differences are displayed as effect sizes with error curves and 95% confidence intervals, two-tailed Mann-Whitney P values are shown for legacy purposes only. Source data are provided as a Source Data file.

Supplementary Figure 4

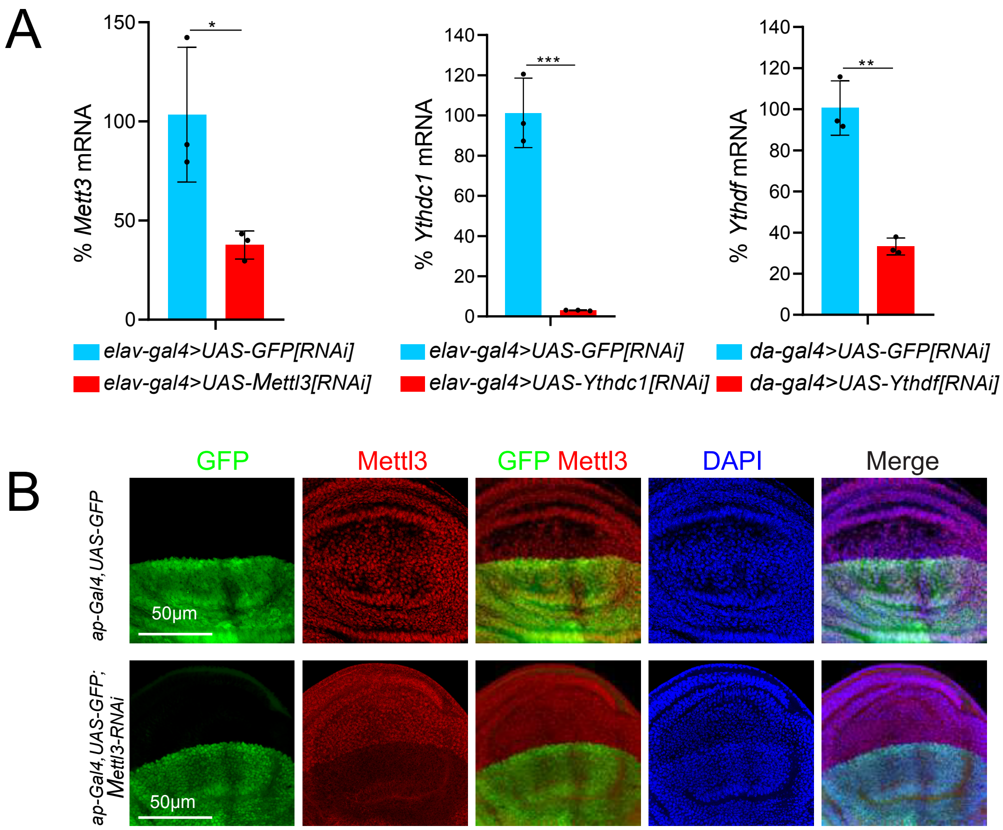

Supplementary Figure 5. Validation of RNAi transgenes against m^{6}A factors.

(A) Validation of *UAS-Mettl3[RNAi]*, *UAS-Ythdc1[RNAi]*, and *UAS-Ythdf[RNAi]* transgenes showing knockdown of cognate targets by qPCR. Error bars, mean ± SD; n = 3 biological replicates. Two-tailed t-test, *p <0.05, **p <0.01, ***p <0.001. *Mettl3[RNAi]* p = 0.0306, *Ythdc1[RNAi]* p = 0.0006, *Ythdf[RNAi]* p = 0.0011. (B) Wing imaginal disc pouch regions carrying *ap-Gal4>UAS-GFP* transgenes, stained for GFP (green) to mark the Gal4/knockdown territory, Mettl3 (red), and DAPI (blue). Top, control disc shows relatively uniform nuclear Mettl3 signals. Bottom, *ap-Gal4>UAS-Mettl3[RNAi]* disc shows specific loss of Mettl3 within the GFP+ dorsal compartment. Source data are provided as a Source Data file.

Supplementary Figure 5

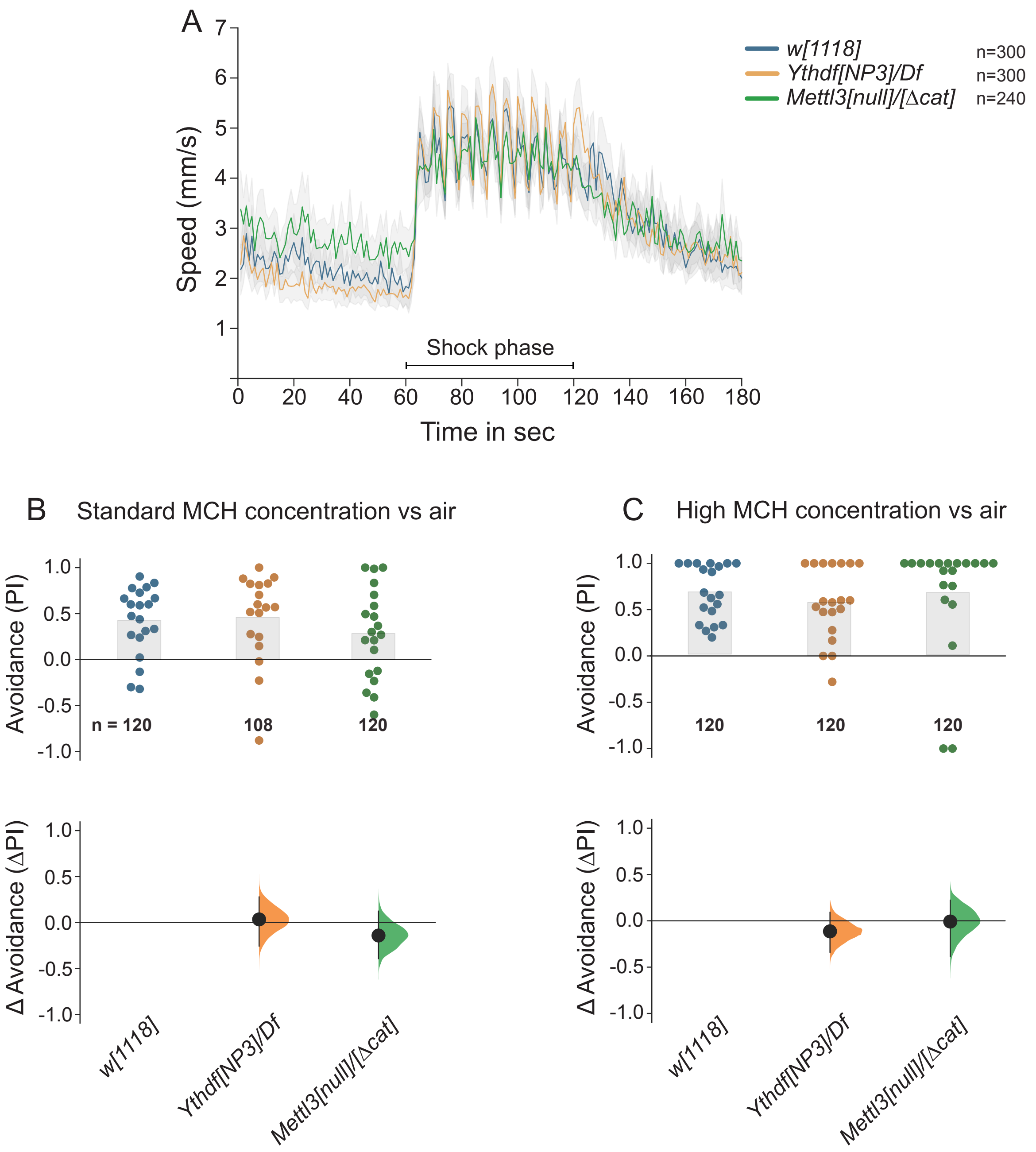

Supplementary Figure 6. m[6]A mutants display unimpaired shock reactivity and olfactory acuity towards a conditioning odor

(A) Activity of control and m6A mutant flies was comparable before, during and after the shock phase. Spikes in activity correspond to each respective shock stimulus given (i.e. 12 shocks in 60 sec). Each line shows the average speed of the respective genotype with a 95% CI error band shown in grey. N = 300 flies for *w[1118]* control, 300 flies for *Ythdf[NP3]/Df* and 240 flies for the *Mettl3[null]/[Δcat]* genotype. (B-C) When given a choice between clean air and MCH in the absence of conditioning, all genotypes preferred the area covered by clean air and avoided the area covered by MCH. This avoidance behaviour was already present at an MCH concentration of about 12ppm (B) and further accentuated when MCH concentration was increased to about 45ppm (C). With both concentrations MCH avoidance was comparable between the controls and m6A mutants suggesting that olfactory acuity of the different genotypes is comparable. (B) *Ythdf[NP3]/Df* vs *w[1118]*: +0.03[95CI -0.26, +0.28], p = 0.6; *Mettl3[null]/[Δcat]*: -0.14[95CI -0.39, +0.13], p = 0.3. (C) *Ythdf[NP3]/Df* vs *w[1118]*: -0.11[95CI -0.34, +0.09], p = 0.5; *Mettl3[null]/[Δcat]*: 0[95CI -0.39, +0.22], p = 0.2. The data were analyzed from the total number of fly shown in the bars and expressed as mean. All control–test differences are displayed as effect sizes with error curves and 95% confidence intervals, two-tailed Mann-Whitney P values are shown for legacy purposes only. Source data are provided as a Source Data file.

Supplementary Figure 6

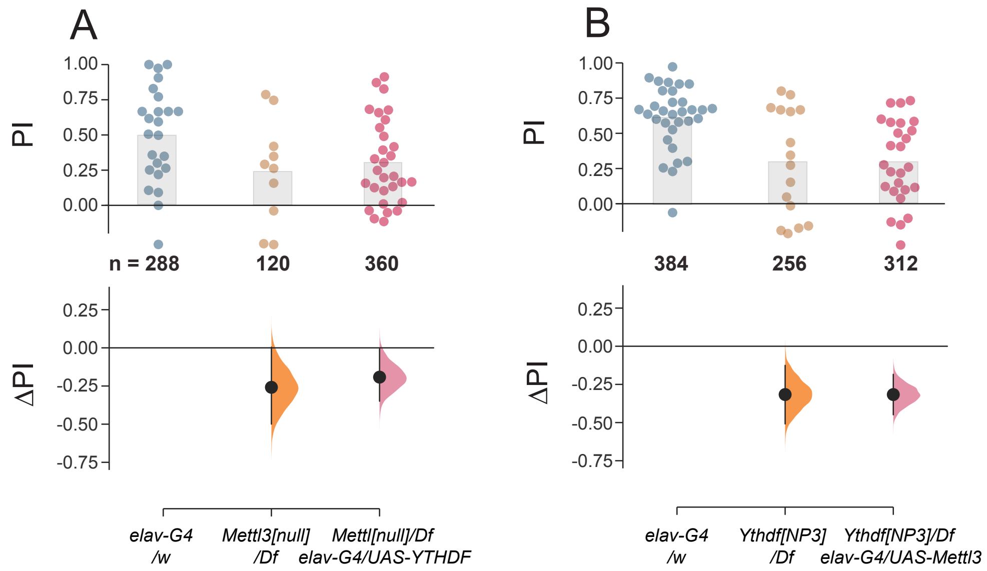

Supplementary Figure 7. No cross-rescue capacity of Mettl3 and Ythdf in STM.

(A) Pan-neuronal expression of Ythdf using *elav-Gal4* (*elav-G4*) did not improve STM impairment in *Mettl3* mutants. *Mettl3[null]/Df* vs *elav-G4/w* = -0.26[95CI -0.5, 0], p = 0.0854. *Mettl3[null]/Df*, *elav-G4/UAS-Ythdf* vs *elav-G4/w* = -0.19[95CI -0.35, 0], p = 0.0302 B: (B) Pan-neuronal expression of Mettl3 in *Ythdf* mutants did not rescue their STM impairment. *Ythdf[NP3]/Df* vs *elav-G4/w* = -0.32[95CI -0.51, -0.13], p = 0.0105. *Ythdf[NP3]/Df*, *elav-G4/UAS-Mettl3* vs *elav-G4/w* = -0.32[95CI -0.45, -0.18], p = 5*10-5. The data were analyzed from the total number of fly shown in the bars and expressed as mean. All control–test differences are displayed as effect sizes with error curves and 95% confidence intervals, two-tailed Mann-Whitney P values are shown for legacy purposes only. Source data are provided as a Source Data file.

Supplementary Figure 7

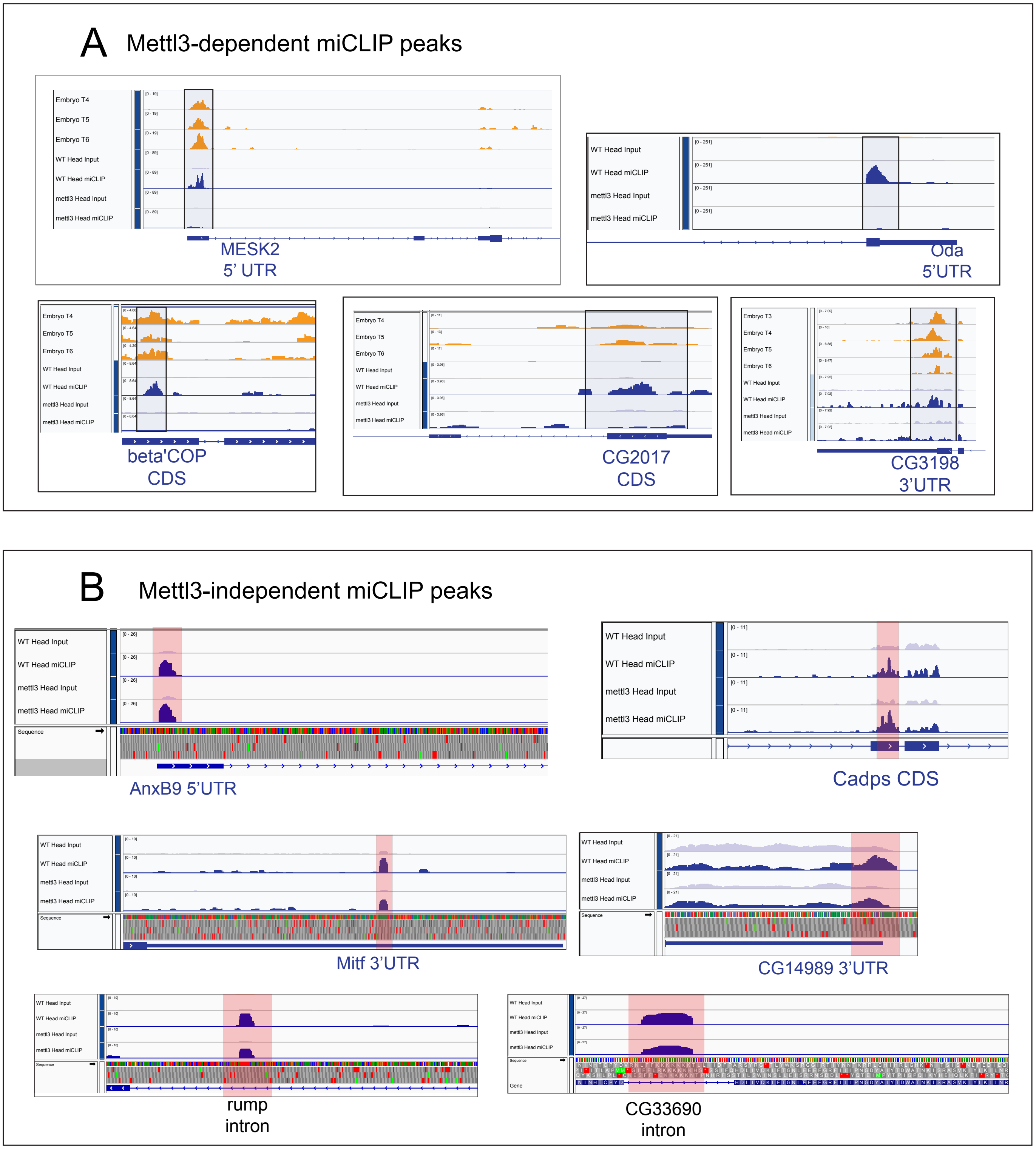

Supplementary Figure 8. Examples of Mettl3-dependent and -independent m^{6}A peaks.

(A) IGV screenshots of several targets displaying Mettl3-dependent miCLIP peaks (highlighted in blue) in various genomic locations (5'UTR, CDS and 3' UTR). Embryo miCLIP data are from Kan et al 2017 and have substantial concordance with our new data at Mettl3-dependent loci even though the tissue types are distinct. (B) IGV screenshots of Mettl3-independent miCLIP peaks (highlighted in red) in various genomic locations (5'UTR, CDS, introns and 3' UTR).

Supplementary Figure 8

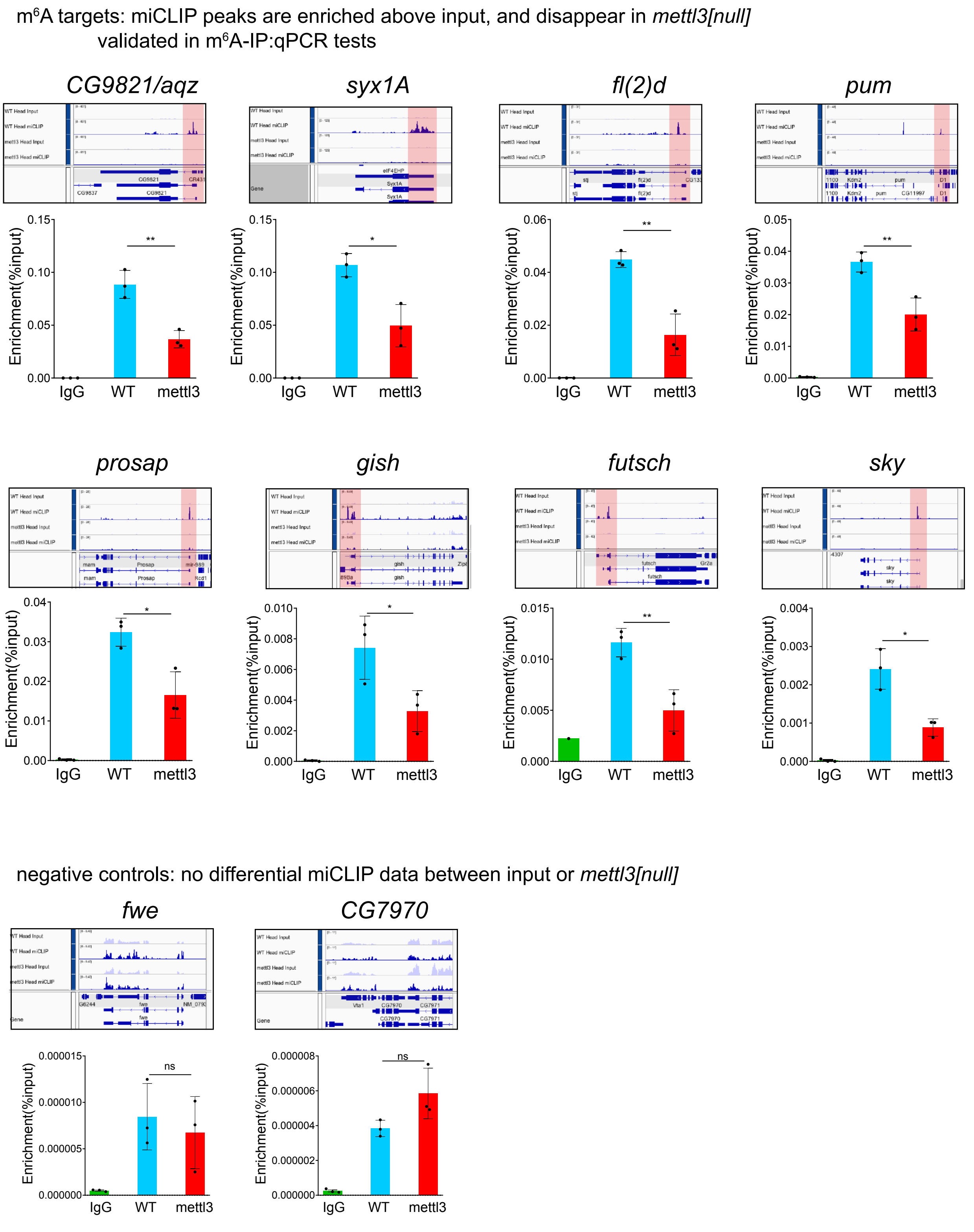

Supplementary Figure 9. m6A target transcripts selected for validation.

Shown are IGV screenshots showing Mettl3-dependent miCLIP peaks (highlighted in pink boxes) at genes selected for m6A-IP validation followed by rt-qPCR; enrichments are shown in IgG control, wild type and Mettl3[null] flies. Negative control genes lacking m6A peaks are shown at bottom. Error bars, mean ± SD; n = 3 biological replicates. Two-tailed t-test, *p <0.05, **p <0.01. syx1A p=0.012, aqz p = 0.0045, fl(2)d p = 0.0043, pum p = 0.0092, prosap p = 0.0160, futsch p = 0.009, gish p = 0.044, sky p = 0.010. n.s. = non-significant, fwe p = 0.604, CG7970 p =0.085. Source data are provided as a Source Data file.

Supplementary Figure 9

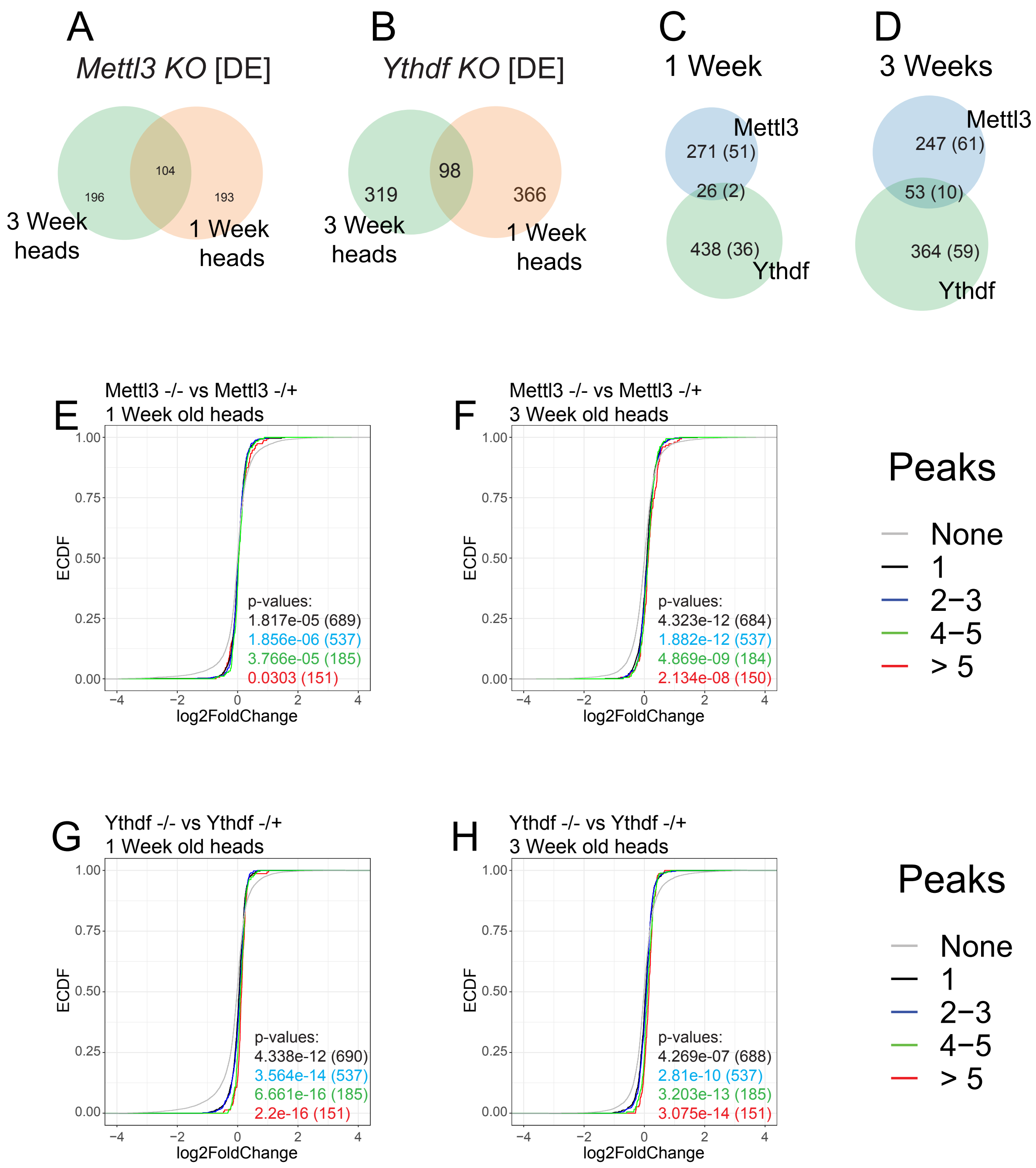

Supplementary Figure 10. m^6A target genes are not differentially expressed (DE) in writer or reader mutants in adult heads.

(A-B) Overlap in consistently differentially expressed genes at 1- and 3-week time points in writer (*Mettl3* - A) or reader (*Ythdf* - B) knockout heads. (C-D) Overlap between consistently DE genes in writer (*Mettl3*) and reader (*Ythdf*) knockout heads at 1-week (C) and 3-week (D) timepoints. Numbers in parentheses are m^6A target genes. (E-H) Empirical cumulative distribution function (ECDF) plots of expression changes in various bins of m^6A targets in writer (E and F) or reader (G and H) mutants at 1-week (E and G) or 3-week (F and H) timepoints. We grouped m^6A target genes based on numbers of peaks per gene, and a bootstrap method was used to generate the background distribution (None) of genes that lacked m^6A peaks. To generate p-values, two-sided Kolmogorov-Smirnov (KS) tests were performed comparing the background distribution and each group of m^6A target genes. Although many of the distributions are significantly different from non-methylated genes, there are no directional changes in expression in any mutant comparison, and all bins of m^6A targets have closely aligned distributions. These data imply there is no overall consistent regulatory impact of m^6A on these targets. Number of targets are included in parentheses.

Supplementary Figure 10

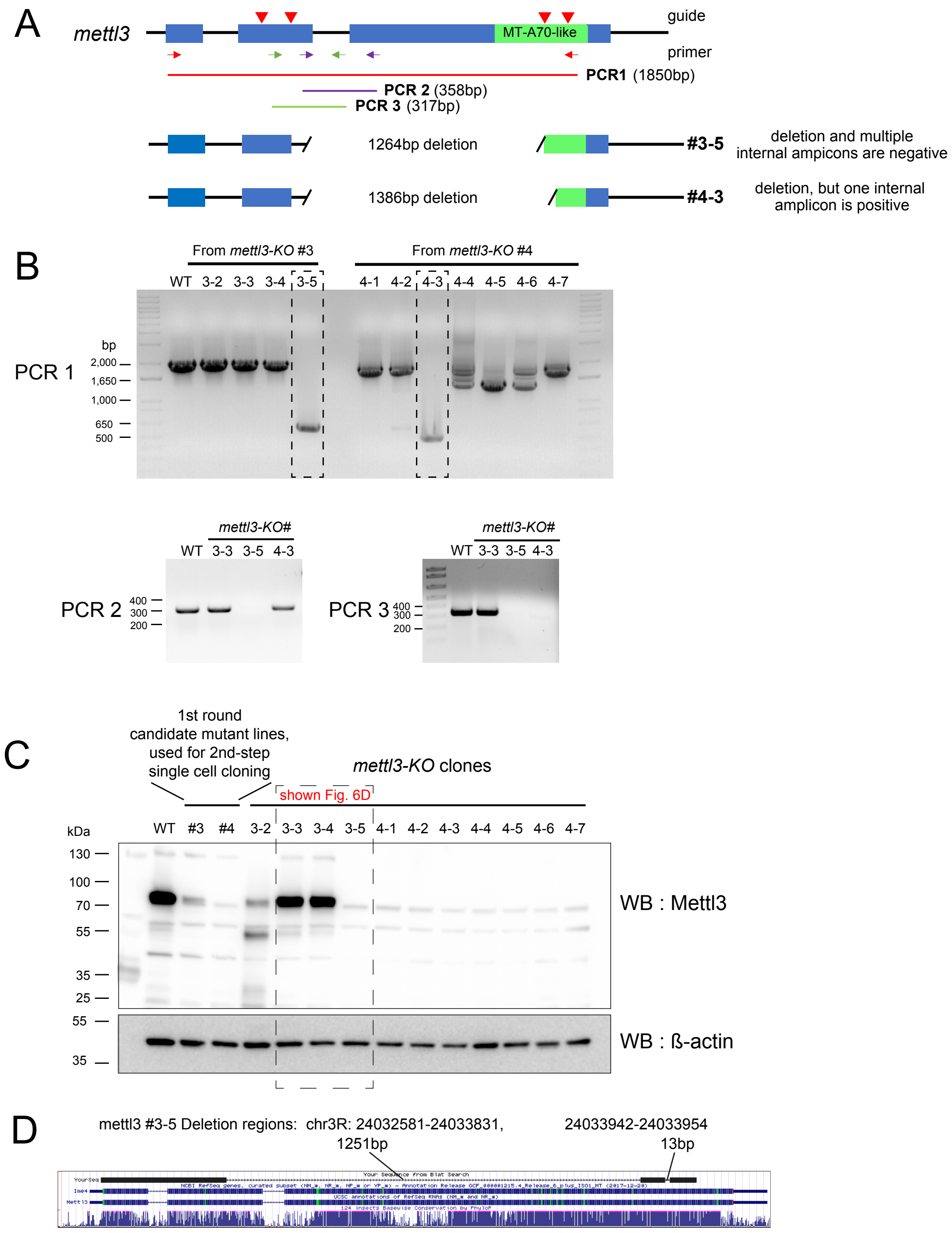

Supplementary Figure 11. Generation of *mettl3-KO* cell lines.

(A) Guide RNAs and multiple amplicons used to genotype Mettl3-KO cell are shown. Two lines with large deletions were isolated, but one of these (#4-3) retained amplification of an internal amplicon even though it was apparently protein null. (B) PCR screening of Mettl3-KO clones. Because S2 cells are difficult to grow clonally, we initially grew limiting dilutions of puro-Cas9/sgRNA cells that survived drug selection with candidate deletions, and then reselected clonal lines with deletions, and further characterized lines #3-5 and #4-3. (C) Western blotting of Mettl3 protein confirms Mettl3-KO clones. This is one representative result from two repeats. (D) Deletion regions of Mettl3-KO #3-5 determined from sequencing; this clone was used for mass spec validation (see main Figure 6D). However, as both #3-5 and #4-3 are null for Mettl3 protein, both were used for functional analyses (see main Figure 6H-I). Source data are provided as a Source Data file.

Supplementary Figure 11
