# Supplementary material for: A neural m6A/Ythdf pathway is required for learning and memory in Drosophila
Source: Nat Commun. 2021 Mar 5;12:1458. doi: 10.1038/s41467-021-21537-1 (PMC7935873; doi:10.1038/s41467-021-21537-1)
Supplement: Supplementary file 3 — Description of Additional Supplementary Files [file 41467_2021_21537_MOESM3_ESM.pdf]

Description of Additional Supplementary Files

Supplementary Data 1.

Proteomics Data The proteomics data file for Figure 1C (searched against Drosophila melanogaster database UniProt (https://www.uniprot.org/).

Supplementary Data 2.

Overview of miCLIP and accompanying datasets Table lists miCLIP and input libraries reported in this study. Counts of pre- and post-map read processing, mutation calling and annotation of CIMs are listed as described in Grozhik et al66.

Supplementary Data 3.

Mettl3-dependent miCLIP peaks Table contains a list of Mettl3-dependent, split peaks. Metadata include enrichment scores, gene segment annotation.

Supplementary Data 4.

CIMs calls from miCLIP head libraries. Table contains the locations of putative single nucleotide m6A sites using the miCLIP CIMs analysis pipeline detailed in Grozhik et al66. Score metadata reflects m/k ratios described in the methods section.

Supplementary Data 5.

Differential gene expression analysis Table lists differentially expressed genes in 1 and 3 week Mettl3-/- and Ythdf-/- fly head libraries. Sheet names detail comparisons and metadata includes fold change and measurements used to determine statistical significance.

Supplementary Data 6.

Oligonucleotide sequences. This file contains the sequences of qPCR primers, genotyping primers, guide RNA, and oligos for making constructs used in the study.
