# Supplementary material for: A neural m6A/Ythdf pathway is required for learning and memory in Drosophila
Source: Nat Commun. 2021 Mar 5;12:1458. doi: 10.1038/s41467-021-21537-1 (PMC7935873; doi:10.1038/s41467-021-21537-1)
Supplement: Supplementary file 10 — Reporting Summary [file 41467_2021_21537_MOESM10_ESM.pdf]

nature research

Corresponding author(s): Eric C. Lai

Last updated by author(s): Mar 20, 2020

Reporting Summary

Nature Research wishes to improve the reproducibility of the work that we publish. This form provides structure for consistency and transparency in reporting. For further information on Nature Research policies, see Authors & Referees and the Editorial Policy Checklist .

Statistics

For all statistical analyses, confirm that the following items are present in the figure legend, table legend, main text, or Methods section.

n/a	Confirmed	
☐	☒	The exact sample size (*n*) for each experimental group/condition, given as a discrete number and unit of measurement
☐	☒	A statement on whether measurements were taken from distinct samples or whether the same sample was measured repeatedly
☐	☒	The statistical test(s) used AND whether they are one- or two-sided<br>*Only common tests should be described solely by name; describe more complex techniques in the Methods section.*
☒	☐	A description of all covariates tested
☐	☒	A description of any assumptions or corrections, such as tests of normality and adjustment for multiple comparisons
☐	☒	A full description of the statistical parameters including central tendency (e.g. means) or other basic estimates (e.g. regression coefficient) AND variation (e.g. standard deviation) or associated estimates of uncertainty (e.g. confidence intervals)
☐	☒	For null hypothesis testing, the test statistic (e.g. *F*, *t*, *r*) with confidence intervals, effect sizes, degrees of freedom and *P* value noted<br>*Give P values as exact values whenever suitable.*
☒	☐	For Bayesian analysis, information on the choice of priors and Markov chain Monte Carlo settings
☒	☐	For hierarchical and complex designs, identification of the appropriate level for tests and full reporting of outcomes
☒	☐	Estimates of effect sizes (e.g. Cohen's *d*, Pearson's *r*), indicating how they were calculated

Our web collection on statistics for biologists contains articles on many of the points above.

Software and code

Policy information about availability of computer code

Data collection	Behavior was recorded with an AVT F-080 Guppy camera (Allied Vision) and video acquisition board (PCI-1409, National Instruments). Western blot record by Image Reader LAS-3000; qPCR CT value was recorded by Biorad CFX96 manager (version 1.10); LAS-AF software was used to acquire images. High through-put sequencing data was collected by illumina HiSeq Control Software v2.2.58 for HiSeq2500 System, as reported in Table S2.
Data analysis	Olfactory preference was measured by tracking the movement of individual flies and scored automatically by using a custom tracking and control program (CRITTA). Behavior data was evaluated using custom Python scripts and the freely available DABEST package as referenced in the manuscript. Bioinformatic analyses utilized a suite of publicly available tools, such as Hierarchical Indexing for Spliced Alignment of Transcripts (HISAT2) and Novoalign (Novocraft Technologies) to map RNA and miCLIP sequencing datasets, as well as tools such as kentUtils and deep. Tools to produce browsable tracks. We used the R bioconductor package DESeq2 to analyze gene expression differences and employed the modified CLIP tool kit pipeline from the Jaffrey lab (PMID: 28349454) to process miCLIP libraries and obtain single nucleotide m6A sites. Additional custom scripts were generated to integrate analyses and develop plots. These are available on the Lai laboratory GitHub page(https://github.com/Lai-Lab-Sloan-Kettering). For western blotting, quantification of each lane or band was carried out by using ImageJ/FIJI software. LAS-AF, was used to overlay images. GraphPad Prism 8.0 was used to make statistical figures.

For manuscripts utilizing custom algorithms or software that are central to the research but not yet described in published literature, software must be made available to editors/reviewers. We strongly encourage code deposition in a community repository (e.g. GitHub). See the Nature Research guidelines for submitting code & software for further information.

Data

Policy information about availability of data

All manuscripts must include a data availability statement. This statement should provide the following information, where applicable:

- Accession codes, unique identifiers, or web links for publicly available datasets
- A list of figures that have associated raw data
- A description of any restrictions on data availability

All STM data sets and python scripts to analyze the data presented in the current study will be made available. High throughput sequencing datasets (m6A-miCLIP and RNA-seq data) reported in this manuscript have been uploaded to the Gene Expression Omnibus (GEO) and are available under accession number GSE147230. RNAseq data from m6A pathway knockdown S2 cells were obtained from GSE79297.
We also integrated reported analyses on mRNA decay and translational efficiency with our m6A profiling. These tables can be found as indicated below:
Burrows et al (Pros>UPRT TU-decay data): https://neuraldevelopment.biomedcentral.com/articles/10.1186/s13064-015-0038-6
Zhang et al (Fly head translational efficiency data): https://journals.plos.org/plosbiology/article?id=10.1371/journal.pbio.2003903

Field-specific reporting

Please select the one below that is the best fit for your research. If you are not sure, read the appropriate sections before making your selection.

☒ Life sciences ☐ Behavioural & social sciences ☐ Ecological, evolutionary & environmental sciences

For a reference copy of the document with all sections, see nature.com/documents/nr-reporting-summary-flat.pdf

Life sciences study design

All studies must disclose on these points even when the disclosure is negative.

Sample size	Sample sizes for STM experiments were determined based on common practices in the field(Cummings G (2013). Understanding the new statistics. Routledge, UK), used at least 120 animals for each analysis. Sample sizes for survival curves were 70-150 flies each genotype. Immunostaining stained 5-10 discs/5-10 brain for each genotypes. qPCR uses 5-10 female fly for each genotypes. For experiments involving quantification of puromycin incorporation, n=4 was chosen as the replicate number. For experiments involving RNA-seq, m6A Mass spec, mRNA decay assay, reporter assay, rip-qPCR, quantification of puromycin incorporation, n=3 was chosen as the replicate number.
Data exclusions	No data were excluded from analysis.
Replication	Each initial STM result was replicated at least once on a separate occasion, combined results were plotted in the manuscript. All attempts at replication of STM results were successful. M6A probe pull-down, RNA-seq, RIP-qPCR, mRNA decay assay, Reporter assay, RIP-qPCR, puromycin incorporation, 1ll these replication attempts were successful. Immunostaining replication attempts were successful, low observed variability between stained samples.
Randomization	The sequence of the genotypes tested in STM studies was randomized on a day to day basis.<br>Discs(L3 larva) and brains (1week old, 3 weeks old) used for imaging were selected randomly for the sex. miCLIP, RNA seq, used female fly head (1week old).
Blinding	Blinding was not relevant for STM experiments because the experiment was recorded and evaluated automatically by the CRITTA software. Experimental manipulation due to implicit bias was not possible without actively tampering with the raw data. Blinding was not possible for the immunostaining, RNA seq, miCLIP libraries making. Since before immunostaining/ libraries making, the cross started with specific genotypes, and need to collect correct genotype fly for staining and extracting RNA.

Reporting for specific materials, systems and methods

We require information from authors about some types of materials, experimental systems and methods used in many studies. Here, indicate whether each material, system or method listed is relevant to your study. If you are not sure if a list item applies to your research, read the appropriate section before selecting a response.

Materials & experimental systems

n/a	Involved in the study	
☐	☒	Antibodies
☐	☒	Eukaryotic cell lines
☒	☐	Palaeontology
☐	☒	Animals and other organisms
☒	☐	Human research participants
☒	☐	Clinical data

Methods

n/a	Involved in the study	
☒	☐	ChIP-seq
☒	☐	Flow cytometry
☒	☐	MRI-based neuroimaging

Antibodies

Antibodies used

Antibodies used in western blotting:
mouse anti-puromycin, DSHB, 2A4, 1:1000
mouse anti-β-tubulin, DSHB, E7, 1:1000
guinea pig anti-Mettl3, Cintia Hongay, 1:5000
rabbit anti-β-actin cell signaling, #4967S, 1:2000
mouse anti-Flag, M2 Sigma-F1804, 1:1000
rabbit α-GFP, Invitrogen, A11122, 1:1000,
Antibodies used in fluorescence:
guinea pig anti-Mettl3, Cintia Hongay, 1:2000
mouse anti-HA, Santa Cruz, sc-7392, 1:1000
Antibody used in miCLIP, and RIP:
rabbit anti-m6A, Synaptic Systems, Cat#202003

Validation

All validation statements can be found on the manufacturer's website.

Eukaryotic cell lines

Policy information about cell lines

Cell line source(s)

S2 cells were obtained from the Drosophila Genome Research Center. The S2-S cell line is a derivative of S2 cells and were a gift from the Stefan Ameres lab. HEK293T cells, obtained from ATCC (ATCC CRL-3216)

Authentication

Cells were genotyped as being male based on Sxl male isoform expression. Mettl3 KO was authenticated by genotyping and antibody staining.

Mycoplasma contamination

All cell lines tested were Mycoplasma were negative.

Commonly misidentified lines
(See ICLAC register)

No commonly misidentified cell lines were used in the stud

Animals and other organisms

Policy information about studies involving animals; ARRIVE guidelines recommended for reporting animal research

Laboratory animals

Drosophila melanogaster (fruit fly). 1 week old female head was used to generate the miCLIP, RNA seq libraries(25oC). 1 week and 3 weeks old fly head was used for brain staining(25oC). L3 larva was used for wingdiscs staining(25oC). 20 day old flies were used for STM and lifespan assay.

Wild animals

No wild animals were used in the study.

Field-collected samples

No field collected samples were used in the study.

Ethics oversight

No approval required for Drosophila melanogaster. The lab is licensed to handle Drosophila

Note that full information on the approval of the study protocol must also be provided in the manuscript.
